# Amorphous calcium carbonate supplementation and bone outcomes in rheumatoid arthritis: A prospective cohort study

**DOI:** 10.1097/MD.0000000000047846

**Published:** 2026-03-06

**Authors:** Wei-Sheng Chen, Po-Ku Chen, Chun-Kai Chen, Hung-Cheng Tsai, Chang-Youh Tsai, Chang-Tei Chou, Shih-Hsin Chang, Chien-Chung Huang, Der-Yuan Chen

**Affiliations:** a Division of Allergy, Rheumatology and Immunology, Taipei Veterans General Hospital, Taipei, Taiwan; b School of Medicine, National Yang-Ming Chiao Tung University, Taipei, Taiwan; c Rheumatology and Immunology Center, China Medical University Hospital, Taichung, Taiwan; d College of Medicine, China Medical University, Taichung, Taiwan; e Department of Food Science, National Taiwan Ocean University, Keelung, Taiwan; f Division of Immunology and Rheumatology, Department of Medicine, Fu Jen Catholic University Hospital and College of Medicine, Fu Jen Catholic University, New Taipei, Taiwan; g PhD Program in Translational Medicine and Rong Hsing Research Center for Translational Medicine, National Chung Hsing University, Taiwan; h Institute of Medicine, Chung Shan Medical University, Taichung, Taiwan.

**Keywords:** amorphous calcium carbonate, bone mineral density, bone turnover markers, osteoporosis, rheumatoid arthritis

## Abstract

Rheumatoid arthritis (RA) is associated with increased risks of osteoporosis and fracture. Amorphous calcium carbonate (ACC) may enhance osteogenic differentiation and increase bone mineral density (BMD). The effects of ACC supplementation on BMD and bone turnover markers (BTMs) in RA have not been explored. This study investigated the influence of ACC supplementation on BMD, BTMs, and the risk of osteoporotic fractures in RA patients. We enrolled 67 RA patients with osteopenia or osteoporosis. BMD was measured by dual-energy X-ray absorptiometry before and after 1 year of ACC supplementation, providing elemental calcium 800 mg/day combined with vitamin D3 400 IU/day. Serum levels of N-terminal propeptide of type I collagen and C-terminal cross-linking telopeptide were measured at baseline and every 3 months following ACC supplementation. The 10-year fracture probability was calculated using the Fracture Risk Assessment Tool (FRAX®), and RA activity was assessed using the 28-joint disease activity score. Multivariate regression analysis revealed that age and corticosteroid dosage ≥5 mg were significant risk factors for major osteoporotic fracture. The 28-joint disease activity scores were inversely correlated with BMD of the right femoral neck (*r* = −0.426, *P* < .001) and left femoral neck (*r* = −0.383, *P* < .005). After 12 months of ACC supplementation, bilateral femoral neck BMD increased significantly (from 0.61 to 0.63 g/cm^2^, both *P* < .001), accompanied by improvement in T-scores. Serum levels of N-terminal propeptide of type I collagen and C-terminal cross-linking telopeptide were significantly decreased (mean 53.42 vs 41.24 ng/mL, *P* < .001; 0.29 vs 0.25 ng/mL, *P* < .05, respectively). Twelve-month ACC supplementation increased bilateral femoral neck BMD and reduced BTM levels in RA patients, particularly in anti-citrullinated peptide antibody–positive patients.

## 1. Introduction

Rheumatoid arthritis (RA), an autoimmune inflammatory disease,^[1]^ is associated with enhanced systemic bone loss and a high risk of hip or vertebral fractures.^[2–4]^ RA-related systemic inflammation and concomitant glucocorticoid (GC) treatment contribute further to an elevated risk of osteoporosis (OP) and osteoporotic fracture.^[5–7]^ The currently available targeted agents for RA include biologic disease-modifying anti-rheumatic drugs (DMARDs) and targeted synthetic DMARDs (tsDMARDs) such as Janus kinase inhibitors (JAKi).^[8–10]^ These b/tsDMARDs therapies may reduce both bone destruction and bone loss.^[11–14]^ Despite the availability of b/tsDMARD therapies today compared to 3 decades ago, an elevated risk of OP and osteoporotic fractures persists in RA,^[15]^ and their management remains suboptimal.

Calcium and vitamin D supplementation are standard supportive regimens in OP management to reduce bone loss and fracture risk. However, the absorption efficiency of conventional calcium salts such as calcium carbonate and calcium citrate is limited by their low solubility, particularly in individuals with reduced gastric acidity or concurrent medication use. Moreover, clinical studies have indicated that bioavailability varies markedly among calcium formulations, influencing therapeutic efficacy and tolerability.^[16,17]^

Amorphous calcium carbonate (ACC) represents a novel, highly soluble form of calcium characterized by its non-crystalline structure and nanoscale particle size, leading to rapid dissolution and superior intestinal absorption compared to crystalline calcium carbonate (CCC).^[18,19]^ Preclinical and clinical studies have demonstrated that ACC supplementation enhances calcium bioavailability and supports bone preservation more effectively than traditional calcium salts. In an ovariectomized rat model of OP, which simulates post-menopausal OP, the use of ACC resulted in superior outcomes compared to other calcium supplements.^[20]^ Vaisman et al^[21]^ also conducted a clinical trial on post-menopausal women and revealed a greater bioavailability of calcium in ACC users compared with CCC supplement users.

To provide mechanistic context, prior studies have compared the skeletal unloading observed in RA-induced immobility to microgravity conditions, both of which disrupt bone metabolism and lead to bone loss. Recent space-based experiments further demonstrated that ACC promotes osteogenic differentiation of human mesenchymal stem cells and increases calcium deposition under microgravity conditions.^[22]^ These findings support ACC’s potential to enhance bone formation in patients with limited mobility, such as those with RA. Given the persistent risk of OP in RA despite advanced disease-modifying antirheumatic drug (DMARD) therapy, evaluating ACC’s role as an adjunctive supplement in this population is warranted.

In this two-center prospective study, we evaluated the effects of ACC supplementation on changes in bone mineral density (BMD), the 10-year probability of osteoporotic fractures, and serum levels of bone turnover markers (BTMs) in RA patients. We also conducted a subgroup analysis to evaluate whether changes in BMD and BTMs after ACC supplementation differ based on seropositivity for anti-citrullinated peptide antibodies (ACPA) or the use of varying doses of GCs.

## 2. Materials and methods

### 2.1. Study participants

This two-center, prospective, single-arm pragmatic intervention study consecutively enrolled eighty patients with RA. Eligibility criteria and exclusions are described above. All patients received Density® (Amorphical Ltd., Israel), an ACC supplement providing 200 mg elemental calcium per tablet, administered as 2 tablets twice daily (total elemental calcium 800 mg/day) and taken with meals. In addition, vitamin D3 was co-administered at a low-dose maintenance level (400 IU/day) in accordance with routine OP care, to support calcium absorption while minimizing the risk of confounding the primary effects of ACC supplementation on bone outcomes. The inclusion criteria were as follows: males older than 65 years or post-menopausalfemales, fulfillment of the 2010 revised criteria of the American College of Rheumatology/EULAR for RA,^[23]^ the presence of either osteopenia or OP as defined by the World Health Organization,^[24]^ a minimum follow-up period of 12 months following ACC supplementation, and ethnically unrelated Han Chinese. Subjects were excluded if they met any of the following criteria: the presence of an active infection or severe hepatic impairment at the time of study entry; voluntary withdrawal from the study despite initial agreement to participate; or current use of hormone replacement therapy. Based on these eligible criteria, a total of 67 patients participated in and completed the present study. All patients received a stable dose of oral GCs, csDMARDs, biologic disease-modifying anti-rheumatic drugs, or tsDMARDs through the 12-month study period. The use of other anti-OP medications (e.g., alendronate, ibandronate, zoledronic acid, raloxifene, denosumab) was prohibited to isolate the effects of ACC on bone metabolism. Adherence was assessed by tablet counts and monthly follow-up visits. Because Density® is a food supplement with a favorable safety profile and manufacturer toxicology reports, no active laboratory safety monitoring was mandated in this trial; future studies will incorporate laboratory safety assessments. RA disease activity was assessed using the 28-joint disease activity score with erythrocyte sedimentation rate (DAS28-ESR).^[25]^ As the study proceeded in an observational, single-arm design without a control group, formal power calculations were not recalculated. Nevertheless, this sample size is considered adequate for exploratory analysis of the changes in BMD and BTMs over time. The results serve as preliminary data for future controlled trials.

### 2.2. The clinical phenotypes

Clinical data collected from the RA patients receiving 12 months of ACC supplementation included demographic data, smoking status, disease duration, disease activity, positivity for rheumatoid factor or ACPA, medication use, and comorbidities.

### 2.3. Bone mineral density evaluation by dual-energy X-ray absorptiometry

BMD measurements of the lumbar spine (LS) (L1–L4) and bilateral femoral neck (FN) were performed at baseline and the end of the study using dual-energy X-ray absorptiometry (Lunar Prodigy, General Electric, Fairfield). BMD was calculated as the bone mineral content divided by the projected bone area (g/cm^2^). The least significant detectable difference was ±0.010 g/cm^2^ for the LS (L1–L4) and ±0.012 g/cm^2^ for the FN. The BMD results at the LS were reported as the mean value of measurements from L1 to L4. T-scores were determined according to the manufacturer’s reference data.

### 2.4. Measurement of 10-year risk of osteoporotic fracture (FRAX® score)

The web-based fracture risk assessment tool (FRAX®) algorithm,^[26]^ specifically the Taiwan FRAX® calculator,^[27]^ was used to calculate the 10-year probability of a major osteoporotic fracture (MOF). This calculator incorporates several clinical risk factors and country-specific fracture and mortality data, including FN BMD, history of prior fractures, parental hip fracture history, age, gender, body mass index, ethnicity, smoking, alcohol use, corticosteroid use, RA, and secondary OP.

### 2.5. Determination of serum levels of BTMs

Levels of BTMs were measured at baseline and months 3, 6, 9, and 12 after initiation of ACC supplementation. N-terminal propeptide of type I collagen; bone formation marker (P1NP) and C-terminal cross-linking telopeptide of type I collagen (CTX); bone resorption marker) were quantified using ECLIA (Modular Roche Diagnostics, Mannheim, Germany). Blood samples were drawn in the morning after overnight fasting to minimize circadian variability.

### 2.6. Statistical analysis

The demographic data are presented as mean ± standard deviation for continuous variables and as number (percentage) for categorical variables. Parameters were tested for normality by the Kolmogorov–Smirnov test. Because BMD of the LS or FN and T-scores were not normally distributed, the correlations between RA disease activity and BTMs or BMD were examined using Spearman rank correlation coefficient. The Mann–Whitney *U* and χ^2^ tests were used to compare continuous and categorical variables in ACPA-positive and ACPA-negative patients. Comparisons of measurements before and after the ACC supplementation were performed using the Wilcoxon signed-rank test. Repeated measurements of BTMs and BMD were analyzed using generalized estimating equations with an identity link, exchangeable working correlation, and robust standard errors to account for within-subject correlation across baseline, 3, 6, 9, and 12 months. Subgroup analyses by ACPA serostatus and GC dose (<5 mg vs ≥5 mg/day) were pre-specified and are reported as exploratory. We report effect sizes with 95% confidence intervals (CIs) and present p-values descriptively. All data were analyzed using the Statistical Package for Social Sciences, version 22.0. A two-sided probability of <.05 was considered statistically significant.

## 3. Results

### 3.1. Comparison of demographic data and laboratory findings between RA patients with and without ACPA

Before starting ACC supplementation, 47 (70.1%) RA patients tested positive for ACPA. As shown in Table 1, ACPA-positive patients had a significantly higher proportion of rheumatoid factor positivity than ACPA-negative patients. ACPA-positive and ACPA-negative groups were comparable at baseline with no significant differences in age, gender, disease duration, DAS28, serum BTMs, BMD, or T-scores (all *P* > .05; see Table 1 for detailed *P* values).

**Table 1:** Comparison of demographic data, bone turnover markers, and bone mineral density between RA patients with and without ACPA.

	ACPA-positive (n = 47)	ACPA-negative (n = 20)	Total RA patients (n = 67)
Age at study entry, yr	67.1 ± 7.8	65.3 ± 9.3	66.5 ± 8.2
Female proportion, n (%)	41 (87.2%)	20 (100.0%)	61 (91.0%)
Body mass index, kg/m^2^	23.25 ± 3.74	23.23 ± 4.90	23.24 ± 4.08
Disease duration (yr)	11.0 ± 2.4	10.9 ± 2.0	11.0 ± 2.3
DAS28 at baseline	3.39 ± 0.71	3.58 ± 0.66	3.44 ± 0.69
RF-positivity, n (%)	38 (80.9%)*	4 (20.0%)	42 (62.7%)
Bone turnover markers			
P1NP (ng/mL)	51.20 ± 27.14	58.65 ± 23.44	53.42 ± 26.14
CTX (ng/mL)	0.28 ± 0.16	0.31 ± 0.14	0.29 ± 0.16
Lumbar spine			
BMD (g/cm^2^)	0.91 ± 0.21	0.86 ± 0.12	0.90 ± 0.19
T-score	−1.86 ± 1.50	−2.08 ± 0.86	−1.93 ± 1.34
Femoral neck, right			
BMD (g/cm^2^)	0.61 ± 0.01	0.60 ± 0.07	0.61 ± 0.09
T-score	−2.70 ± 0.58	−2.63 ± 0.41	−2.68 ± 0.54
Femoral neck, left			
BMD (g/cm^2^)	0.61 ± 0.09	0.60 ± 0.08	0.61 ± 0.09
T-score	−2.70 ± 0.53	−2.60 ± 0.56	−2.67 ± 0.54
Total hip, right			
BMD (g/cm^2^)	0.73 ± 0.08	0.71 ± 0.08	0.72 ± 0.08
T-score	−2.06 ± 0.66	−1.99 ± 0.81	−2.04 ± 0.71
Total hip, left			
BMD (g/cm^2^)	0.71 ± 0.09	0.70 ± 0.10	0.71 ± 0.09
T-score	−2.15 ± 0.70	−2.05 ± 0.84	−2.12 ± 0.74
Glucocorticoid use, n (%)	22 (46.8%)	8 (40.0%)	30 (44.8%)
Glucocorticoid (mg/d)	2.18 ± 2.74	2.25 ± 3.33	2.20 ± 2.90
csDMARDs			
Methotrexate, n (%)	13 (27.7%)	5 (25.0%)	18 (26.9%)
Hydroxychloroquine, n (%)	27 (57.5%)	8 (40.0%)	35 (52.2%)
Salazopyrine, n (%)	6 (12.8%)	3 (15.0%)	9 (13.4%)
bDMARDs, n (%)	20 (42.6%)	8 (40.0%)	28 (41.8%)
JAKi, n (%)	20 (42.6%)	6 (30.0%)	26 (38.8%)
Hypertension, n (%)	7 (14.9%)	3 (15.0%)	10 (14.9%)
Diabetes, n (%)	2 (4.3%)	1 (5.0%)	3 (4.5%)
Smoking, n (%)	3 (6.4%)	1 (5.0%)	4 (6.0%)

Data were expressed as mean ± SD or number (%).

ACPA = anti-citrullinated peptide antibodies, bDMARDs = biologic DMARDs, BMD = bone mineral density, csDMARDs = conventional synthetic disease-modifying anti-rheumatic drugs, CTX = C-terminal cross-linking telopeptide of type I collagen, DAS28 = the 28-joint disease activity score, JAKi = Janus kinase inhibitors, P1NP = N-terminal propeptide of type I collagen, RA = rheumatoid arthritis, RF = rheumatoid factor, SD = standard deviation.

* *P* <.001, versus ACPA-negative patients, as determined by the Student *t* test.

### 3.2. Logistic regression analysis for predicting a high risk of MOF before ACC supplementation

As illustrated in Table 2, the univariate analysis identified age, the use of GC, and daily GC dosage of ≥5 mg as potential predictors of a high risk of MOF (*P* <.05). After adding the variables with *P*-values <0.3 in the univariate analysis, multivariate analysis revealed that age at study entry (odds ratio: 1.19; 95% CI: 1.08–1.30, *P* <.001) and a daily GC dosage of ≥5 mg (odds ratio: 8.17; 95% CI: 1.59–42.0, *P* <.05) were significant predictors of a high risk of MOF before ACC supplementation.

**Table 2:** Logistic regression analysis of demographic data, clinical characteristics, and laboratory data for predicting the FRAX-calculated high risk of major osteoporotic fracture in RA patients before supplement with amorphous calcium carbonate.

Baseline variables	Univariate model			Multivariate model		
	OR	95% CI	*P* value	OR	95% CI	*P* value
Age at study entry, yr	1.15	1.06–1.25	<.001	1.19	1.08–1.30	<.001
Gender						
Male	Ref.					
Female	0.77	0.13–4.54	.771			
BMI	1.07	0.94–1.21	.333			
RF positivity	0.63	0.22–1.77	.379			
ACPA positivity	1.44	0.50–4.18	.498			
PINP baseline levels	0.98	0.96–1.00	.108			
CTX baseline levels	0.37	0.02–8.52	.537			
The use of glucocorticoids	3.47	1.20–10.0	.022			
**Daily glucocorticoids ≧5 mg**	**3.52**	**1.02–12.1**	**.046**	**8.17**	**1.59–42.0**	**.012**
bDMARD	1.25	0.46–3.41	.660			
JAKi monotherapy	1.30	0.46–3.70	.625			
Methotrexate	0.53	0.18–1.59	.258			
Hydroxychloroquine	0.70	0.26–1.88	.477			
Statin use	2.70	0.29–25.6	.386			
Comorbidities						
Hypertension	2.70	0.29–25.6	.386			
Current smoker	0.51	0.04–6.01	.594			

A high-risk major osteoporotic fracture is defined as the 10-yr probability of fracture higher than 20% using the Taiwanese Fracture Risk Assessment Tool (FRAX®) calculator.

Variables in multivariate model (backward: condition; *P* value cutoff <.3): Age, PINP baseline levels, the use of glucocorticoids, daily dose of glucocorticoids, and methotrexate. Statistically significant differences are indicated in bold.

ACPA = anti-citrullinated peptide antibodies, bDMARDs = biologic DMARDs, BMI = body mass index, CI = confidence interval, CTX = C-terminal cross-linking telopeptide of type I collagen, FRAX = fracture risk assessment, JAKi = Janus kinase inhibitors, OR = odds ratio, P1NP = N-terminal propeptide of type I collagen, RA = rheumatoid arthritis, RF = rheumatoid factor.

### 3.3. Correlation between RA activity and BMD or BTMs before ACC supplementation

RA disease activity, reflected by the DAS28-ESR score, was inversely correlated with BMD at the right FN (FN-BMD) (correlation coefficient, *r* = −0.426, *P* <.001) and left FN-BMD (*r* = −0.383, *P* <.005). Otherwise, RA disease activity was not significantly correlated with LS BMD (LS-BMD, *r* = −0.223, *P* = .070) or serum levels of any BTMs, including P1NP and CTX.

### 3.4. Changes in BMD, T-score, the FRAX-calculated risk of osteoporotic fractures, and BTMs in RA patients after ACC supplementation

As shown in Table 3, in the entire cohort, bilateral femoral-neck BMD increased significantly (right: 0.61 → 0.63 g/cm^2^, *P* <.001; left: 0.61 → 0.63 g/cm^2^, *P* <.001). This overall improvement was accompanied by modest T-score increases. Subsequent subgroup analyses showed that these gains were more pronounced in ACPA-positive patients, but directionally similar in both subgroups.

**Table 3:** The change in BMD, the FRAX-calculated probability of osteoporotic fracture, and bone turnover markers levels in RA patients receiving one-year ACC supplementation.

	Before ACC	After ACC	*P*-value
Lumbar spine			
BMD (g/cm^2^)	0.90 ± 0.19	0.91 ± 0.20	.1356
T-score	−1.93 ± 1.34	−1.88 ± 1.45	.2326
Femoral neck, right			
BMD (g/cm^2^)	0.61 ± 0.09	0.63 ± 0.10	<.0001***
T-score	−2.68 ± 0.54	−2.51 ± 0.67	<.0001***
Femoral neck, left			
BMD (g/cm^2^)	0.61 ± 0.09	0.63 ± 0.09	<.0001***
T-score	−2.67 ± 0.54	−2.55 ± 0.47	.0013**
Hip FRAX	16.45 ± 17.66	15.80 ± 17.91	.5457
MOF FRAX	28.60 ± 18.24	27.43 ± 18.36	.0733
P1NP (ng/mL)	53.42 ± 26.14	41.24 ± 18.81	<.0001***
CTX (ng/mL)	0.29 ± 0.17	0.25 ± 0.15	.0271*

Data were expressed as mean ± SD.

ACC = amorphous calcium carbonate, BMD = bone mineral density, CTX = C-terminal cross-linking telopeptide of type I collagen, FRAX = fracture risk assessment, MOF = major osteoporotic fracture, P1NP = N-terminal propeptide of type I collagen, RA = rheumatoid arthritis, SD = standard deviation. Statistically significant differences are indicated in bold.

* *P* <.05.

** *P* <.01.

*** *P* <.001, determined by Wilcoxon signed rank.

Serum levels of BTMs, including P1NP and CTX, were significantly decreased after 12 months of ACC supplementation (mean, 53.42 ng/mL vs 41.24 ng/mL, *P* <.001 for P1NP; 0.29 ng/mL vs 0.25 ng/mL, *P* <.05 for CTX). In the dynamic change (Fig. S1, Supplemental Digital Content, https://links.lww.com/MD/R467, Quarterly changes in serum P1NP and CTX during ACC supplementation, n = 67, 64, 60, 59, 57 at baseline, months 3, 6, 9, 12, respectively). Shaded bands represent 95% CIs, serum levels of bone-resorption marker CTX were markedly reduced by 13.8% early in month 3 and maximally declined by 21.1% in month 6 during ACC supplementation. Serum levels of bone-formation marker P1NP also declined significantly by 9.6% early in month 3, then increased by 7.6% in month 12 compared to month 9 levels during ACC supplementation.

### 3.5. Differential effects of ACPA on changes in BMD and BTMs

**Figure 1.**
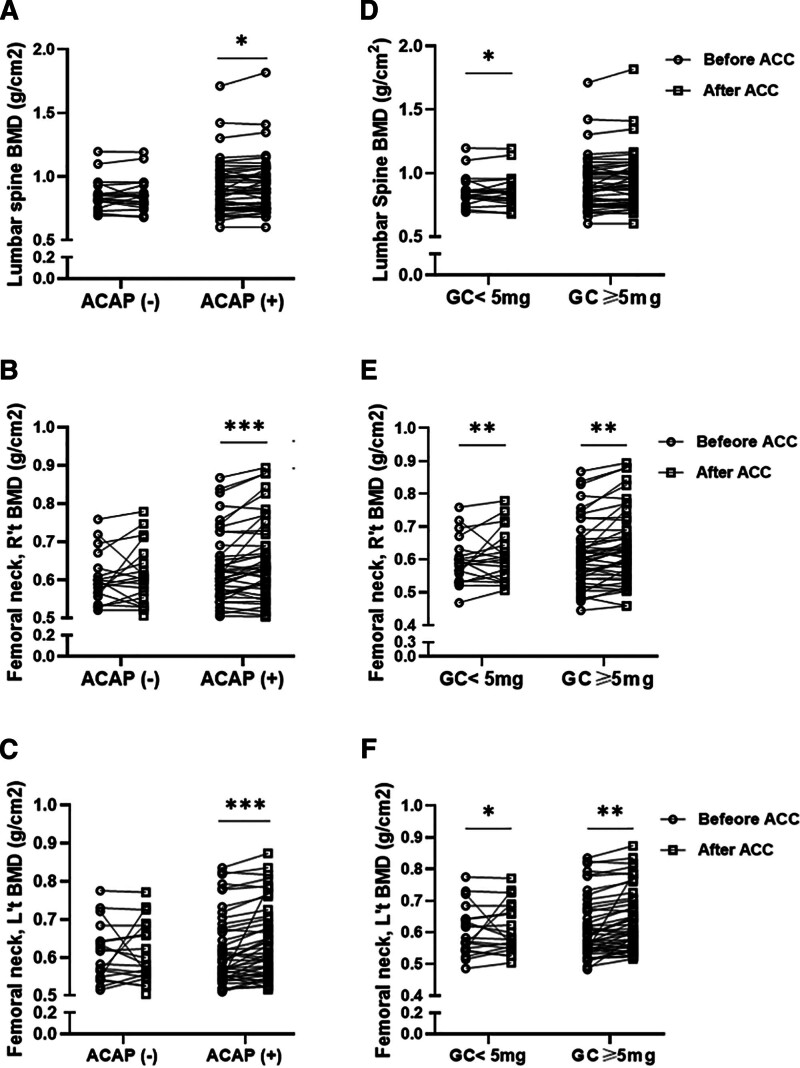
The effects of ACPA or glucocorticoids dose on the changes of BMD after ACC supple-mentation in RA patients. The changes of (A) lumbar spine-BMD; (B) right femoral neck-BMD, and (C) left femoral neck-BMD in ACPA-negative and ACPA-positive patients with RA. The changes in (D) lumbar spine-BMD; (E) right femoral neck-BMD, and (F) left femoral neck-BMD in RA patients receiving a daily glucocorticoid dose of ≥5 mg and dose of <5 mg. *P value < .05, **P value < .005, ***P value < .001, by the Wilcoxon signed rank test. ACPA = anticitrullinated protein antibody, ACC = amorphous calcium carbonate, BMD = bone mineral density, RA = rheumatoid arthritis.

We further categorized RA patients into ACPA-positive and ACPA-negative subgroups to investigate whether changes in BMD and BTMs after ACC supplementation differ based on ACPA seropositivity. As illustrated in Table 4 and Figure 1, changes in lumbar-spine and femoral-neck BMD before and after 12-month ACC supplementation. Each line connects paired baseline and post-treatment values (n = 67). Median change and 95% CI are shown; sensitivity analysis excluding outliers yielded consistent results. We found a significant increase in LS-BMD and bilateral FN-BMD exclusively in ACPA-positive patients (0.91 ± 0.21 vs 0.93 ± 0.22, *P* <.05 for LS-BMD; 0.62 ± 0.10 vs 0.64 ± 0.11, *P* <.001 for right FN-BMD; 0.61 ± 0.09 vs 0.64 ± 0.10, *P* <.001, for left FN-BMD). Overall, serum CTX decreased significantly in the full cohort (0.29 → 0.25 ng/mL, *P* = .027). However, when analyzed within ACPA subgroups, these decreases did not reach statistical significance (both *P* > .10). This indicates that the overall decline was driven by combined data rather than by either subgroup alone.

**Table 4:** The change in BMD and bone turnover markers levels in RA patients with and without ACPA after one-year ACC supplementation.

	Before ACC	After ACC	*P*-value
P1NP (ng/mL)			
ACPA (−)	58.65 ± 23.44	43.31 ± 15.46	.014*
ACPA (+)	51.20 ± 27.14	40.36 ± 20.15	.002**
CTX (ng/mL)			
ACPA (−)	0.31 ± 0.14	0.26 ± 0.17	.133
ACPA (+)	0.28 ± 0.16	0.24 ± 0.14	.115
Lumbar spine BMD (g/cm^2^)			
ACPA (−)	0.86 ± 0.12	0.86 ± 0.13	.588
ACPA (+)	0.91 ± 0.21	0.93 ± 0.22	.027*
Femoral neck, R’t BMD (g/cm^2^)			
ACPA (−)	0.60 ± 0.07	0.61 ± 0.08	.182
ACPA (+)	0.62 ± 0.10	0.64 ± 0.11	<.001***
Femoral neck, L’t BMD (g/cm^2^)			
ACPA (−)	0.60 ± 0.08	0.61 ± 0.08	.716
ACPA (+)	0.61 ± 0.09	0.64 ± 0.10	<.001***

ACC = amorphous calcium carbonate, ACPA = anti-citrullinated peptide antibodies, BMD = bone mineral density, CTX = C-terminal cross-linking telopeptide of type I collagen, P1NP = N-terminal propeptide of type I collagen, RA = rheumatoid arthritis.

Wilcoxon signed rank.

* *P* <.05.

** *P* <.01.

*** *P* <.001.

### 3.6. Differential effects of glucocorticoid dose on changes in BMD and BTMs after ACC supplementation

Because BMD and BTMs may respond differently to daily GC usage, we divided patients into 2 subgroups: those who received a daily GC dose of ≥5 mg and those receiving a daily dose of <5 mg. As illustrated in Table 5 and Figure 1D–F, bilateral FN-BMD increased significantly in all patients after 12 months of ACC supplementation, whereas LS-BMD increased significantly only in those who received a daily GC dose of <5 mg (0.89 ± 0.15 vs 0.90 ± 0.15, *P* <.05). As shown in Table 5, serum P1NP levels decreased significantly, but no significant change in serum CTX levels after ACC supplementation was observed in any patients receiving GC therapy.

**Table 5:** The change in BMD and BMTs levels in RA patients receiving daily glucocorticoid (GC) dose of ≥5 mg and those receiving daily dose of glucocorticoid of <5 mg after one-year ACC supplementation.

	Before ACC	After ACC	*P*-value
P1NP (ng/mL)			
GC <5 mg	55.41 ± 27.54	42.46 ± 19.62	.001**
GC ≥5 mg	48.75 ± 22.48	38.36 ± 16.86	.015*
CTX (ng/mL)			
GC <5 mg	0.29 ± 0.17	0.25 ± 0.16	.071
GC ≥5 mg	0.28 ± 0.12	0.23 ± 0.13	.216
Lumbar spine BMD (g/cm^2^)			
GC <5 mg	0.89 ± 0.15	0.90 ± 0.15	.025*
GC ≥5 mg	0.92 ± 0.25	0.92 ± 0.28	.471
Femoral neck, Rt BMD (g/cm^2^)			
GC <5 mg	0.61 ± 0.09	0.63 ± 0.11	.003**
GC ≥5 mg	0.60 ± 0.09	0.61 ± 0.09	.006**
Femoral neck, Lt BMD (g/cm^2^)			
GC <5 mg	0.61 ± 0.09	0.62 ± 0.09	.010*
GC ≥5 mg	0.61 ± 0.09	0.63 ± 0.10	.002**

ACC = amorphous calcium carbonate, BMD = bone mineral density, BMTs = bone turnover markers, CTX = C-terminal cross-linking telopeptide of type I collagen, GC = glucocorticoid, P1NP = N-terminal propeptide of type I collagen, RA = rheumatoid arthritis.

Wilcoxon signed rank.

* *P* <.05.

** *P* <.01.

## 4. Discussion

This study demonstrated that higher RA disease activity was modestly associated with lower femoral-neck BMD, consistent with previous reports linking systemic inflammation to bone loss. The observed effect sizes (Spearman *r* = –0.38 to –0.43) indicate a moderate inverse relationship rather than a strong causal link. Multivariate regression analysis showed that age and concomitant use of GCs at a dose of ≥5 mg were significant predictors of a high MOF risk in RA patients. The bilateral femoral-neck BMD increased by approximately 3 to 4% over 12 months, a magnitude that falls within the range reported in some early-phase anti-resorptive therapy trials conducted in populations with low bone mass or osteopenia.^[28–31]^ However, given the observational nature of this prospective cohort, these findings should be interpreted with caution, as residual confounding from disease activity, concomitant medications, and lifestyle-related factors cannot be fully excluded. Therefore, causal inference cannot be made based on the current study design. However, the FRAX-estimated 10-year fracture risk did not significantly change, likely because FRAX integrates age and clinical risk factors, which remain stable over one year. These results suggest that short-term BMD improvement from ACC may precede measurable changes in fracture probability. In the subgroup analysis, an improvement in LS-BMD and FN-BMD was observed only in ACPA-positive patients. Serum levels of BTMs, including P1NP and CTX, also declined significantly after ACC supplementation. To our knowledge, we are the first to reveal that ACC supplementation has beneficial effects on bilateral FN-BMD in elderly men and post-menopausal women with RA, particularly ACPA-positive patients. Although GC use may have a deleterious impact on BMD, we still observed an add-on benefit of ACC supplements in RA patients receiving a specific dose of GCs for disease management.

The first step to help improve bone health for RA patients is to identify those at high risk of developing osteoporotic fractures. In the present study, age and concomitant use of corticosteroids ≥5 mg/day were significant predictors of MOF in RA patients. Age is a parameter expected to influence MOF risk because it is integral to the definition of FRAX. Glucocorticoid-induced OP and osteoporotic fracture are well-known phenomena, and a meta-analysis indicates a strong association between GC use and low LS-BMD.^[28]^ Previous studies have revealed that osteoporotic fracture risks during GC therapy appeared dose-dependent.^[6,32]^ Some studies have also demonstrated that low-dose GCs may inhibit inflammation, thereby counteracting their harmful effects on bone metabolism.^[33]^ In our study, concomitant GC use at doses of ≥5 mg/day was a significant risk factor for MOF in RA patients. Given the conflicting results on the impact of GC dosage on osteoporotic fracture risk in RA patients, our findings require further validation.

Accumulating evidence indicates the multifactorial nature of OP development in RA, including RA-related inflammation, aging, medication use, and immobility.^[34,35]^ Inflammation contributes to bone loss in RA patients, as higher disease activity or more severe disease is associated with a greater risk of bone loss.^[36]^ Resonating with these observations, an inverse correlation between DAS28-ESR scores and bilateral FN-BMD was observed in our RA patients. Despite the availability of effective b/tsDMARD therapies,^[8–10]^ RA patients remain at an elevated risk of OP and osteoporotic fractures.^[15]^ Additional supplementary therapies may provide beneficial effects for OP in RA patients.

RA is characterized by persistent synovitis, bone destruction, functional disability, and substantial immobility.^[1]^ Immobility is one of the 5 criteria proposed as an accurate tool for identifying OP in RA patients.^[37]^ RA patients with immobility exhibit similarities to individuals experiencing microgravity conditions. Growing evidence suggests that microgravity may induce changes in bone metabolism, leading to a decline in bone density.^[38–40]^ Recently, ACC has emerged as a promising candidate for preventing these detrimental effects under microgravity conditions.^[22]^ Cohen et al^[22]^ conducted 2 studies aboard the International Space Station. They found that ACC treatment promotes mesenchymal stem cell differentiation into osteoblasts, enhancing bone formation and increasing calcium deposits.^[22]^ Besides, accumulating evidence suggests that the increased bioavailability of ACC is strongly linked to its nanometric particle size and amorphous phase, resulting in higher dissolution rates than CCC forms.^[20,21]^ Herein, this is the first to assess the effects of 12-month ACC supplementation on BMD in elderly men and post-menopausal women with RA. The increased BMD of bilateral FNs observed in our study suggests that ACC supplementation may help improve bone density in RA patients with osteopenia or OP.

Given the negative impact of ACPAs on BMD in patients with early and established RA,^[41,42]^ we assessed whether changes in BMD following ACC supplementation differ based on ACPA seropositivity. Our results showed a significant increase in BMD at the LS and FNs exclusively in ACPA-positive patients, consistent with previous findings where BMD improvement after two-year tocilizumab therapy was observed only in ACPA-positive RA patients.^[12]^ Our findings also align with a previous study showing that the adoptive transfer of ACPAs into mice induces osteoclastogenesis.^[43]^ These observations support the hypothesis that autoantibodies, such as ACPA, may influence bone metabolism. However, our findings should be validated in larger cohorts of RA patients.

Although GC therapy is often associated with bone loss, ACC supplementation appeared to maintain or improve BMD even among patients receiving low- to moderate-dose GCs. As no formal interaction between ACC use and GC dose was tested, this finding should be interpreted as exploratory and not as evidence of an “add-on” or synergistic effect. Future controlled studies are warranted to confirm whether ACC may help stabilize bone metabolism under chronic GC exposure.

Although GC use negatively affects bone health in RA patients,^[32]^ conflicting results have been reported regarding the impact of GC dosage.^[6,32,33]^ Lodder et al^[44]^ found that GC use was not independently associated with BMD in RA patients. A randomized trial showed that adding 10 mg of GCs to an MTX-based tight control strategy did not lead to bone loss in RA patients receiving bisphosphonates, an anti-osteoporotic therapy.^[45]^ All these findings, including ours, challenge the traditional concept of the harmful effects of GCs on bone metabolism in RA. Nevertheless, the 2021 American College of Rheumatology guidelines recommend minimizing GC use due to their adverse effects.^[46]^

BTMs have been suggested as useful tools for monitoring the effectiveness of OP therapy and predicting fracture risk.^[47]^ Serum levels of CTX (a marker of bone resorption) and P1NP (a marker of bone formation) are commonly used as BTMs. In our study, serum levels of P1NP and CTX decreased significantly after 12 months of ACC supplementation, indicating a reduction in overall bone turnover. Our results resonate with findings that the suppression of BTMs by potent anti-resorptive agents, such as bisphosphonates, correlates with stabilization or even an increase in BMD in osteoporotic patients.^[48]^ As illustrated in Figure S1 (Supplemental Digital Content, https://links.lww.com/MD/R467), the dynamic changes in BTMs in our patients showed that serum CTX and P1NP levels declined significantly by month 6, with the decline in CTX-a marker of bone resorption-being more pronounced than the suppression of P1NP. Since BMD reflects the net balance between bone formation and resorption, greater suppression of bone resorption relative to bone formation during ACC supplementation can lead to an overall increase in BMD. The increases in BMD are consistent with the BTM results, suggesting that bone loss was halted after 12 months of ACC supplementation.

This study is the first to reveal the supplementary benefits of ACC on bilateral FN-BMD and to report the differential effects of ACPAs and GC dosage on bone density after ACC supplementation. We also establish a link between disease activity and BMD in RA patients.

However, our study has several limitations. First, baseline serum 25-hydroxyvitamin D [25(OH)D] levels were not systematically measured in this pragmatic cohort, which limits our ability to assess the potential modifying role of vitamin D status on the observed bone outcomes. Future controlled studies should incorporate baseline and longitudinal 25(OH)D measurements. Second, all subjects in this study are of Chinese ethnicity, and therefore, our findings may not be generalizable to other ethnic groups. Although the International Osteoporosis Foundation has recommended serum P1NP and CTX levels as reference BTMs, their levels may fluctuate due to circadian rhythms, food intake, or RA disease activity.^[49]^ To minimize the potential influence of circadian rhythms, serum samples for BTMs measurement were collected in the morning after overnight fasting. Third, RA patients taking other calcium supplements, such as CCC forms, were not included as a control group for comparative analysis, limiting our ability to determine the superiority of ACC supplementation. Fourth, the study lacks a control group of biologically naïve patients with comparable RA activity, which may lead to an underestimation of the impact of b/tsDMARDs beyond ACC supplementation on bone metabolism. However, no significant difference was observed in BMD changes between our patients with and without concomitant b/tsDMARDs. Finally, the primary goal of OP treatment is to reduce fragility fractures; however, their incidence remains low over a short follow-up period. Future large-scale, multi-center studies are required to validate the supplementary benefit of ACC in RA patients receiving different DMARDs therapies.

## 5. Conclusions

Our study revealed the 12 months of ACC supplementation led to a significant increase of bilateral FN-BMD and a parallel reduction in BTMs in RA patients. Age and daily GC dose ≥ 5 mg emerged as exploratory predictors of higher fracture probability, underscoring the multifactorial nature of skeletal fragility in RA. Although the FRAX-estimated 10-year fracture risk did not change, this finding likely reflects the tool’s limited sensitivity to short-term BMD gains; maintaining or modestly increasing BMD over one year may still help stabilize bone health in this population. Larger, controlled studies are warranted to confirm the durability and clinical relevance of these effects.

## Acknowledgments

The Amorphous Calcium Carbonate supplement was supported by Universal Integrated Corp., Taipei, Taiwan. The authors also thank Shiow-Jiuan Wey, MD, of the Chung Shan Medical University Hospital, Taiwan, for manuscript and English editing.

## Author contributions

**Conceptualization:** Wei-Sheng Chen, Chun-Kai Chen, Chang-Youh Tsai, Der-Yuan Chen.

**Data curation:** Wei-Sheng Chen, Po-Ku Chen, Chun-Kai Chen, Hung-Cheng Tsai, Shih-Hsin Chang, Der-Yuan Chen.

**Formal analysis:** Po-Ku Chen, Chun-Kai Chen, Hung-Cheng Tsai, Shih-Hsin Chang, Chien-Chung Huang, Der-Yuan Chen.

**Investigation:** Wei-Sheng Chen, Chang-Youh Tsai, Chang-Tei Chou, Chien-Chung Huang, Der-Yuan Chen.

**Supervision:** Chun-Kai Chen, Der-Yuan Chen.

**Writing – original draft:** Chun-Kai Chen, Der-Yuan Chen.

**Writing – review & editing:** Der-Yuan Chen.

## Supplementary Material


