# Supplementary material for: Amorphous calcium carbonate supplementation and bone outcomes in rheumatoid arthritis: A prospective cohort study
Source: Medicine (Baltimore). 2026 Mar 6;105(10):e47846. doi: 10.1097/MD.0000000000047846 (PMC12975258; doi:10.1097/MD.0000000000047846)

**Figure S1.** Serum levels of BTMs, including P1NP and CTX, were significantly decreased after 12 months of ACC supplementation.


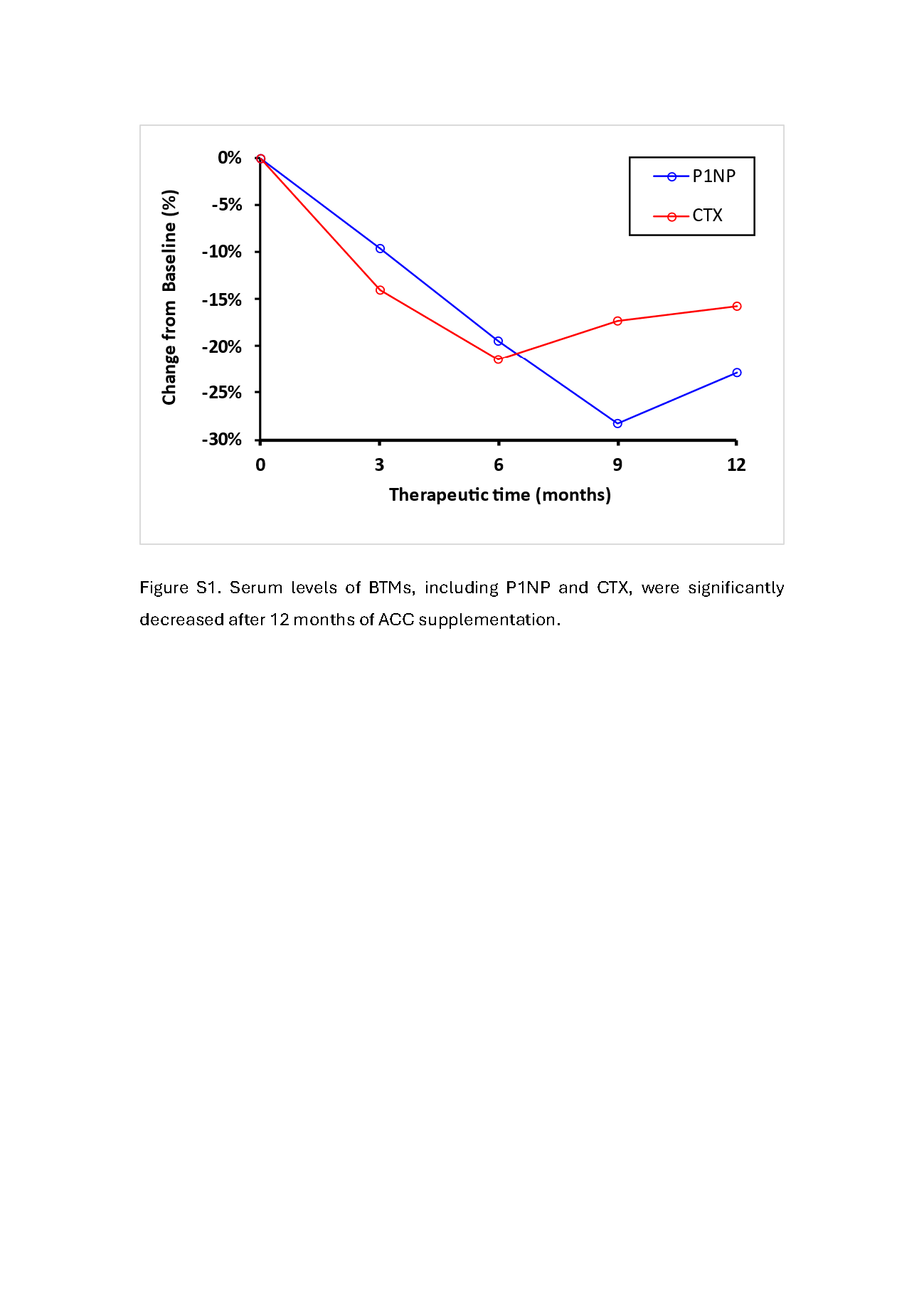

Supplement: Supplementary file 1 [file medi-105-e47846-s001.docx]
